# -216G/T (rs712829), a functional variant of the *EGFR* promoter, is associated with the pleural metastasis of lung adenocarcinoma

**DOI:** 10.3892/ol.2013.1442

**Published:** 2013-07-03

**Authors:** HAISHENG GUO, YUNHUI XING, RUIBAO LIU, SHAOPING CHEN, XIA BIAN, FANG WANG, CHUNMEI YANG, XUNGUO WANG

**Affiliations:** 1 Department of Oncology, Dongying People’s Hospital, Dongying, Shandong 257091, P.R. China; 2 Department of Tuberculosis, Shengli Hospital of Shengli Oilfield, Dongying, Shandong 257075, P.R. China

**Keywords:** *EGFR*, gene polymorphism, pleural metastasis, lung adenocarcinoma

## Abstract

Numerous mutations and variants in the epidermal growth factor receptor (*EGFR)* gene have been demonstrated to be associated with the occurrence, metastasis and prognosis of various types of tumors, including lung cancer. Thus, the present study aimed to investigate whether -216G/T (rs712829), a functional polymorphism of the *EGFR* promoter that is able to induce *EGFR* activation and overexpression, is associated with the pleural metastasis of lung adenocarcinoma. The study subjects were comprised of 326 patients with primary lung adenocarcinoma and 312 matched cases with pleural metastasis. The -216G/T genotypes were determined in all subjects by PCR amplification and direct DNA sequencing, and *EGFR* expression was also evaluated by immunohistochemical staining in the primary tumor tissues with various -216G/T genotype backgrounds. The results showed that the frequencies of allele T and genotypes G/T and T/T in the pleural metastasis group were significantly higher compared with those in the non-metastasis group, with adjusted ORs of 1.46 (95% CI, 1.015–1.963) for G/T and 1.97 (95% CI, 1.051–3.152) for T/T. Furthermore, the expression of the EGFR protein was higher in the primary lung adenocarcinoma tissues with -216T/T and -216G/T compared with those with -216G/G (P<0.05). These results collectively indicate that the -216G/T polymorphism in the EGFR promoter is associated with the risk of the pleural metastasis of lung adenocarcinoma and that this effect may be associated with -216G/T-induced overexpression of the EGFR protein.

## Introduction

Lung cancer is mostly diagnosed at an advanced stage and is the leading cause of mortality caused by malignancies worldwide (1,2). Of all types of lung tumors, non-small cell lung cancer (NSCLC) accounts for ~80%, with adenocarcinoma as the most prevalent subtype (3,4). Furthermore, NSCLC, particularly lung adenocarcinoma, often presents with pleural metastasis and even malignant pleural effusion (MPE) at the time of diagnosis, which is an indicator of poor prognosis for patients (1). Although the pleural metastasis of lung adenocarcinoma occurs with high frequency and indicates a poor prognosis, the underlying genetic mechanism remains largely unclear.

Epidermal growth factor receptor (EGFR), a transmembrane glycoprotein with tyrosine kinase activity, is a major regulator of several signaling pathways (5). EGFR activation and/or overexpression often leads to signal transduction cascades, which in turn contribute to cell proliferation, angiogenesis, cancer invasion and metastasis (6). In humans, EGFR is frequently overexpressed in 50–81% of NSCLC and such overexpression has been demonstrated to be associated with cancer susceptibility, metastasis, survival prognosis and chemotherapy response (7–14). Numerous mutations and variants in the *EGFR* gene have also been characterized in human lung tumors, of which a number have been demonstrated to be associated with EGFR overexpression or activation (7–9). EGFR mutations, which are mostly limited to the first four exons, occur more often in lung cancer patients with adenocarcinoma histology, Asian origin, female gender and a non-smoking background (13,15). Additionally, several functional variants in the EGFR gene, including CA-SSR1 (CA repeat in intron 1 of EGFR), -216G/T and R497K, have also been detected with higher frequency in lung cancer, as well as other tumors, and these variants often result in increased promoter activity and EGFR transcription (16–18). Therefore, it has been proposed that genetic alterations in the EGFR gene may be associated with the development and metastasis of lung cancers (11,12,19).

-216G/T (rs712829), a functional polymorphism in the *EGFR* promoter, is located in the Sp1 recognition site where multiple protein factors and transcriptional start sites have been identified (20–22). Since the Sp1 binding site is a region that is critical for the regulation of *EGFR* transcription (23–25), the replacement of G by T at position -216 increases promoter activity by 30%, thereby resulting in a higher *EGFR* expression level (18,22,26). In clinical studies, it has been shown that -216G/T may be associated with inherited susceptibility to cancers, as well as other common diseases (22,27). Furthermore, studies have also observed that -216G/T was able to predict drug response and that the NSCLC patients with at least one -216T allele exhibited significant improvements with regard to the effects of gefitinib treatment on survival time (28,29). Although evidence indicates that -216G/T may be correlated with the development, treatment response and survival prognosis of lung cancer patients, its role in cancer metastasis remains largely unknown.

Based on previous findings, we proposed that -216G/T in the *EGFR* promoter may be associated with an increased risk of the pleural metastasis of lung adenocarcinoma. Therefore, in the present study, -216G/T genotyping and immunohistochemical detection of *EGFR* protein expression was performed in two cohorts of patients with primary lung adenocarcinoma and pleural metastasis respectively, with the aim of determining the association between -216G/T variants in the *EGFR* gene and the risk of the pleural metastasis of lung adenocarcinoma.

## Materials and methods

### Patient information

A total of 638 patients, including 326 cases of primary lung adenocarcinoma and 312 matched cases with pleural metastasis, were enrolled into the study between May 2008 and April 2011 at Shandong Provincial Hospital (Shandong, China). All the subjects enrolled in the study were at stage IV according to the revised TNM staging system for NSCLC announced by the International Association for the Study of Lung Cancer (IASLC) (30). The diagnoses for all the patients, including that of pleural metastasis, were confirmed by pathological and/or cytological examination. The clinical information from these patients, including age, gender, smoking history, cancer stage and pathology/cytology examination result, was recorded. The enrolled patients were categorized into smokers and those who had never smoked according to their smoking history. The detailed characteristics of all the patients are listed in Table I. This study was approved by the institutional review board of Shandong Provincial Hospital and informed consent was obtained from all patients.

### Sample preparation

Peripheral blood samples were collected consecutively from all the enrolled patients and tissue samples were also obtained from a number who had received a bronchoscopic biopsy during diagnosis. All blood samples were retained for the genotyping of genetic variants in the EGFR gene and the tissues for evaluating EGFR expression. The peripheral blood was collected into EDTA-coated tubes and DNA was extracted with a commercial DNA extraction kit (Keyuan Biotechnology Development Center, Beijing, China) according to the manufacturer’s instructions. Tissue samples were routinely fixed in 10% buffered formalin and embedded in paraffin for diagnosis and the examination of EGFR protein expression.

### -216G/T genotyping

PCR applications of the -216G/T variants in the *EGFR* gene were performed with the forward, 5′-GCTTGGTCCTCTTCGGCATCT-3′ and reverse, 5′-CCGTCTTGACCAGTCGCTTA-3′ primers. The PCR reaction was set up in a 50 μl volume containing 25 μl Master Mix (Tiangen Biotech Company, Beijing, China), 2 μl forward and reverse primers, 25 ng/4 μl DNA template and 19 μl nuclease-free water. PCR reactions were run with the following cycling conditions: pre-denaturation at 94°C for 5 min, denaturation at 94°C for 30 sec, annealing from 68 to 60°C decreasing at 1°C/cycle for 8 cycles and at 59°C for 30 cycles, extension at 72°C for 30 sec and a final extension for 7 min, with a total of 38 cycles. The PCR products were sequenced directly in the sense and antisense directions using an ABI373 instrument (Applied Biosystems, Foster City, CA, USA).

### Immunohistochemical staining

Paraffin-embedded tissues were subjected to immunohistochemical staining with EGFR antibody using a streptavidin-biotin immunoperoxidase kit (BioGenex, Fremont, CA, USA) according to the manufacturer’s instructions. In brief, following antigen retrieval and blocking of endogenous peroxidase activity, tissue slides (5 μm) were incubated with EGFR monoclonal antibody (Santa Cruz Biotechnology, Inc., Santa Cruz, CA, USA) at a 1:500 dilution, overnight at 4°C in a moist chamber. Subsequent to being washed in PBS, the slides were sequentially incubated with the secondary antibody for 45 min at room temperature, stained with diaminobenzidine tetrahydrochloride (DAB) and finally counterstained with hematoxylin. Staining without the primary antibody was employed to create a negative control.

The level of EGFR expression was evaluated by multiplying the positive cell rate and staining intensity, as reported in previous studies (31,32). In brief, positive cell rates of 0, 1–10, 11–50 and 51–100% were scored as 0, 1, 2, 3 and 4, respectively, while staining intensity grades of 0, 1, 2 and 3 referred to negative, weak positive, moderately positive and markedly positive staining for EGFR, respectively, as described previously (33). EGFR expression was assessed by two independent investigators who were blinded to the clinical data. Discrepancies were solved by discussion.

### Statistical analysis

All statistical analyses were performed using the SPSS 10.0 statistical software package (SPSS, Inc., Chicago, IL, USA). The categorical variables were analyzed using the χ^2^ test and Fisher’s exact test, as appropriate. Odds ratios (ORs) and their 95% confidence intervals (CIs) were estimated and adjusted by logistic regression analysis for the clinicopathological factors. EGFR expression data were analyzed statistically with the Mann-Whitney U test. A two-sided value of P<0.05 was considered to indicate a statistically significant difference.

## Results

### Clinical characteristics of the patients

In total, 638 patients were enrolled in the present study, including 326 cases of primary lung adenocarcinoma and 312 matched cases with pleural metastasis. The characteristics of the enrolled subjects are presented in Table I. Between the primary lung adenocarcinoma and metastatic groups, the distributions of clinicopathological factors were not significantly different (Table I). The χ^2^ test showed that the genotype distribution of -216G/T was in agreement with the Hardy-Weinberg equilibrium (P>0.05) in the two groups.

### Genotype/allele frequencies of -216G/T

**Figure 1:**
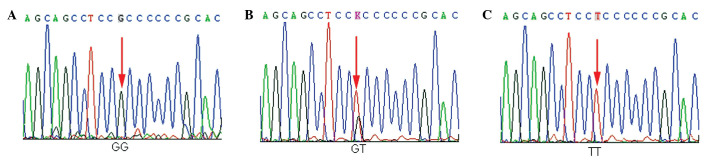
Representative sequencing wave figures for -216 G/T. Three genotypes (A) G/G, (B) G/T and (C) T/T in *EGFR* are shown, with each variant base indicated by a red arrow. EGFR, epidermal growth factor receptor.

The three genotypes of -216 G/T in *EGFR*, namely G/G, G/T and T/T, were detected among the subjects from the Chinese population. Each genotype is demonstrated by a representative sequencing wave figure in Fig. 1. The genotype and allele frequencies of -216G/T in primary lung adenocarcinoma and pleural metastasis are described in Table II. The minor allele, T, was detected in 32.85% of the chromosomes in patients with pleural metastasis, which was a significantly higher rate than the 23.93% in patients with primary lung adenocarcinoma (OR, 1.56; 95% CI, 1.217–1.989; P=0.000; Table II). Similarly, the genotype frequencies of GT and TT in pleural metastasis were significantly higher compared with those in primary lung adenocarcinoma, with ORs of 1.39 (95% CI, 1.003–1.994) and 1.46 (95% CI, 1.015–1.963), respectively (Table II). Furthermore, following adjustment for the clinicopathological variables using logistic regression analysis, the adjusted ORs were 1.46 (95% CI, 1.015–1.963) for G/T and 1.97 (95% CI, 1.051–3.152) for T/T (Table II).

### EGFR expression

**Figure 2:**
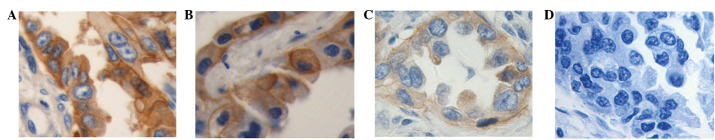
Representative immunohistochemical images indicating staining patterns of EGFR in lung adenocarcinoma tissues with various genotypes. Immunohistochemical staining for EGFR in primary lung adenocarcinoma specimens with (A) T/T, (B) G/T and (C) G/G genotypes and staining of the the negative control in (D) T/T specimens with EGFR antibody replaced by phosphate-buffered saline. The brown staining indicates a positive result for EGFR protein expression in A, B and C, while D shows a lack of staining. All images were obtained with a 40X objective lens. EGFR, epidermal growth factor receptor).

It has been demonstrated experimentally that -216G/T variants result in *EGFR* activation and thereby increased EGFR expression, suggesting that there may be potential differences in EGFR expression among individuals with the various -216G/T genotypes. In line with such a concept, EGFR expression was assessed in the present study in the primary lung adenocarcinoma tissues of various -216G/T genotypes by immunohistochemical staining. The immunohistochemical staining was performed in the tumor tissues of primary lung adenocarcinoma, which included 21 cases with the G/G genotype, 22 cases with the G/T genotype and 19 cases with the T/T genotype. As shown in Fig. 2, the diffuse/intense brown staining represented the positive expression of the EGFR protein. The EGFR expression scores were 10 for the G/T genotype and 7 for the T/T genotype, each being significantly higher than the score of 3 for the G/G genotype (P<0.05).

## Discussion

Although a number of mutations/variants in the *EGFR* gene have been demonstrated to be associated with the development and metastasis of lung cancer (9,10), it remains largely unclear whether -216G/T, a functional variant in the *EGFR* promoter, has any critical role in the pleural metastasis of lung adenocarcinoma. In the present study, the -216G/T genotypes of G/T and T/T were detected in patients with pleural metastasis at higher frequencies compared with cases of primary lung adenocarcinoma, and the expression of the EGFR protein was also increased significantly in the former group compared with the latter. All these results collectively indicate that -216G/T is associated with the pleural metastasis of lung adenocarcinoma, possibly by affecting EGFR overexpression.

To the best of our knowledge, the present study demonstrates for the first time that 216G/T and T/T are associated with an increased risk for the pleural metastasis of lung adenocarcinoma. Several other studies, although different in certain aspects from the present study of germline -216G/T variants, have also documented the associations of somatic mutations in *EGFR* with pleural metastasis of lung cancer. One study reported that the rate of somatic mutations in *EGFR* was significantly higher in lung cancer patients with pleural metastasis compared with patients without metastasis (68.4 vs. 50.5%) (34). Another study observed that somatic mutations of *EGFR* were discordant between primary tumors and corresponding pleural metastases in a significant portion of lung adenocarcinomas, although the mutation frequency was higher in primary lesions compared with pleural metastases (35). The reasons for such contrary results remain unknown at present. More recently, a study noted that de4 *EGFR,* a novel *EGFR* variant with aberrant splicing of exon 4, exhibited a higher level of metastasis promoting activity in comparison with the wild-type (36). Therefore, together, the evidence from the present and previous studies suggests that *EGFR* mutations/variants may be involved in the process of the pleural metastasis of lung cancer, although with certain inconsistencies between various studies.

-216G/T is located in the essential region of the *EGFR* promoter and the G to T allele transition at this loci leads to increased *EGFR* transcription by causing the binding of Sp1 and promoter activity (22,24). Therefore, EGFR expression was examined in lung adenocarcinoma patients with various genotypes in order to further clarify the potential molecular mechanism underlying the pleural metastasis associated with the -216G/T variants. The present study showed that T/T and G/T were associated with an increase in EGFR expression compared with G/G, indicating that -216G/T variants may contribute, at least partially, to the promoter activity and thereby the variability of EGFR expression in lung tumor cells. Thus, it is rational to deduce that EGFR overexpression due to -216G/T variants is likely to promote the pleural metastasis of lung cancer. Several other studies have also indicated a critical role for EGFR overexpression in the metastasis of lung adenocarcinoma. Clinical studies have shown that the elevated serum levels or overexpression of the EGFR protein were associated with the aggressiveness and metastasis of NSCLC (37). Moreover, EGFR overexpression due to polymorphisms has been observed in several other types of tumors, including breast and gastrointestinal cancer (29,34–36), and EGFR inhibitors were able to inhibit the metastasis and invasiveness of tumor cells, including lung cancers, even at a low dose that had no significant effect on primary tumor growth (38,39). Therefore, the majority of clinical studies have demonstrated that the pleural metastasis of lung cancer was closely associated with the overexpression of EGFR, although certain others have reported contrary results (40). Consistent with the majority of clinical studies, experimental studies have also shown that the activation of the EGFR pathway was likely to be involved in the process of cancer metastasis (41), while EGFR overexpression promoted the metastasis of several types of tumor cells (42–44).

Ethnic differences in the distributions of *EGFR* mutations and polymorphisms have been identified between Asian and Caucasian individuals and are considered to be responsible for the ethnic differences in clinical responses to EGFR inhibitor treatment (45–48). Asian ethnicity is known to be a predictor of a good clinical response to EGFR inhibitors and is associated with a high incidence of *EGFR* mutations (45,46). Similarly, ethnic differences are also evident in the frequency of -216G/T variants. Previous studies have reported that the heterogeneous -216G/T and the minor allele, T, were common in African American and Caucasian populations, but less frequent in Asian individuals (18,22). In the present study, a low frequency of T/T was observed in a Chinese population, similar to studies reported in Asian populations, which included Chinese individuals (18,49).

The present findings may have certain clinical implications. *EGFR* mutations are now attractive targets for the treatment and prevention of lung cancer. Studies have shown that somatic mutations in the EGFR tyrosine kinase domain are associated with an advanced stage, poor prognosis, survival outcome and clinical response of NSCLC to EGFR inhibitors (50–52). Thus, -216G/T, a germline variant loci, may also contribute to the variability in biological characteristics and treatment response to EGFR inhibitors and could be used as a predictive biomarker. However, it should be noted that in contrast to the majority of somatic mutations reported previously, the clinical implications of this less frequent variant of -216 G/T remain largely unknown and require further investigation.

In conclusion, the present study demonstrated for the first time that the -216G/T polymorphism in the *EGFR* promoter is a genetic susceptibility factor for the pleural metastasis of lung adenocarcinoma in a Chinese population, with the T allele and G/T and T/T genotypes being associated with increased metastatic risk. Additional studies are required to confirm these conclusions in other populations due to the evident ethnic differences with regard to *EGFR* mutations/variants.

## Figures and Tables

**Table I:** Clinical characteristics of the patients.

Clinicopathological parameters	Primary lung adenocarcinoma (n=326)		Pleural metastasis (n=312)		P-value^a^
	
	No. of cases	%	No. of cases	%	
Age (years)					0.335
<60	191	58.5	171	54.8	
≥60	135	41.5	141	45.2	
Gender					0.473
Male	166	50.9	150	48.1	
Female	160	49.1	162	51.9	
Smoking status					0.583
Never	180	55.2	179	57.4	
Smoker	146	44.8	133	42.6	
Differentiation grade					0.434
Well	36	11.0	42	13.5	0.225
Moderate	143	43.9	122	39.1	
Poor	147	45.1	148	47.4	

a Two-sided χ^2^ test.

**Table II:** Association of -216G/T genotype/allele frequencies in EGFR with the risk of pleural metastasis of lung adenocarcinoma.

	Primary lung adenocarcinoma (n=326)		Pleural metastasis (n=312)			
				
Genotype/allele	n^a^	%	n^a^	%	OR (95% CI)	Adjusted OR (95% CI)^b^
Genotype						
GG	194	59.51	146	46.79		
GT	108	33.13	127	40.71	1.39 (1.003–1.914)	1.46 (1.015–1.963)
TT	24	7.36	39	12.50	1.80 (1.054–3.067)	1.97 (1.051–3.152)
Allele						
G	496	76.07	419	67.15		
T	156	23.93	205	32.85	1.56 (1.217–1.989)	

a n refers to patient number for the genotype and chromosome number for the allele;

b OR was adjusted for age, gender, smoking status and differential grade of tumor cells.

OR, odds ratio; 95% CI, 95% confidence interval; EGFR, epidermal growth factor receptor.
